# Analysis of Negative Reviews of Orthopedic Oncology Surgeons: An Investigation of Reviews from Healthgrades, Vitals, and Google

**DOI:** 10.1155/2022/4351427

**Published:** 2022-12-10

**Authors:** Leeann Qubain, Evan H. Richman, Vincent Eaton, Joseph C. Brinkman, Krista M. Goulding

**Affiliations:** ^1^University of Arizona College of Medicine–Phoenix, Phoenix, AZ, USA; ^2^Department of Orthopedic Surgery, University of Colorado, Denver, CO, USA; ^3^Creighton University School of Medicine–Phoenix Regional Campus, Phoenix, AZ, USA; ^4^Department of Orthopedic Surgery, Mayo Clinic, Phoenix, AZ, USA

## Abstract

**Background:**

Physician review websites (PRWs) are increasing in usage and popularity. Our purpose is to characterize one-star reviews of orthopedic oncology surgeons to understand factors in healthcare that contribute to patient satisfaction.

**Methods:**

Orthopedic oncology surgeons were randomly selected from the Musculoskeletal Tumor Society. A search for one-star reviews was performed on Google Reviews, Healthgrades, and Vitals.com. Reviews were classified as clinical or nonclinical. Statistical analyses were performed regarding the frequency of reviews and complaints for each category.

**Results:**

Of the 7,733 reviews discovered, 908 (11.7%) were identified as one-star reviews. Of 907 usable complaints, 362 (40.8%) were clinical and 545 (59.2%) were nonclinical. The most common nonclinical complaints included bedside manner (65%) and limited time with providers (19%). The most common clinical complaints included complications (26%) and disagreements with the treatment plan (26%). There were 120 surgical and 221 nonsurgical reviews. Surgical patients had a higher rate of clinical complaints. Nonsurgical patients had a higher rate of total complaints.

**Conclusion:**

To the best of our knowledge, this is the first study examining PRWs regarding orthopedic oncology surgeons. Most one-star reviews were due to nonclinical complaints from nonsurgical patients. The most common factors are bedside manner, limited time with provider, phone communication issues, and rude/unprofessional conduct.

## 1. Introduction

As technology continues to integrate into healthcare, there is a progressive increase in virtual and online medical resources [1]. One particular aspect of this movement toward online resources includes physician review websites (PRWs), which have grown increasingly popular in recent years [2]. Specifically, it was reported in 2017 that 39% of patients utilize these resources [3]. These platforms also hold value for patients, as it has been reported that 60% of Americans believe online reviews of physicians are either somewhat or very important in helping them seek out healthcare services [2]. Further, over 50% of patients using online reviews have eliminated specific healthcare providers from their list of options based on the content of online reviews [4].

The emergence of online review sites has been encouraged by the policy in addition to patient preference. The changes mandated by the Affordable Care Act of 2010 created a shift toward patient-centered and value-based outcomes in medicine. In collaboration with patient empowerment, an update by Consensus Core Set of Orthopedic Measures (CCSOMs) was released to simplify value-based measurements [5]. One principle outlined in the CCSOM was “patient experience,” which prioritized patients' self-reported outcomes over more traditional objective measures of health outcomes. To incentivize providers to meet specific patient quality benchmarks, hospitals have implemented reimbursement models that heavily weigh these subjective patient experiences, launching a new era of value-based care physician compensation. This is commonly measured and reported with the publicly available Hospital Consumer Assessment of Healthcare Providers and Systems (HCAHPS) Surveys, which use a standardized survey instrument and data collection methodology that is used to measure patients' perspectives of hospital care. With the above in mind, it has become increasingly clear that patients' opinions are becoming more valuable.

Despite their popularity, the utility of these patient-created reviews is still highly controversial [6–9]. Understandably, as these reviews affect compensation, patient-physician relationships, and potential patients, physicians have voiced concerns about the validity of these reviews [10]. Despite the controversy, it is vital that physicians have a thorough understanding of their online reviews and what may lead to a poor online reputation. While there are prior studies analyzing orthopedic surgeons' online ratings, few have examined the reasons for negative reviews. In Bernstein's review, most of the studies included that evaluated PRWs of various orthopedic specialties analyzed presence of positive reviews and average physician scores [11]. In addition, there have been no studies investigating reviews or ratings of orthopedic oncologists [11, 12]. The aim of this study is to characterize one-star reviews of orthopedic oncology surgeons to further understand factors that affect the patient experience in this unique patient group. This information will allow orthopedic oncology surgeons to further understand what factors are most considered by patients and identify targets to improve their online reputation.

## 2. Methods

A random number generator was utilized to select 200 of the 265 surgeons listed on the Musculoskeletal Tumor Society (MSTS) “Member Search” tool on the MSTS website [13]. We decided on 200 surgeons as we believed this would capture an appropriate sample size of reviews. A search was then performed for all reviews listed under the MSTS surgeon's name on the following websites: Google Reviews, Healthgrades, and Vitals.com. These three websites were chosen as they have been listed by the reputation industry as the most important for physicians to consider [14]. Only one-star reviews were collected; all other reviews (out of a possible 5 stars) were excluded from this study. In addition, for each of the three websites, a surgeon's average rating (1–5), number of reviews, and number of 1-star reviews were recorded.

One-star reviews were then classified by two separate authors (LQ and VE) as either clinical or nonclinical. Nonclinical reviews included comments referring to physician professionalism, midlevel professionalism, front-desk professionalism, time with provider, wait time, facilities, cost, billing issues, phone communication, or scheduling issues. Clinical reviews included comments with reference to complications, readmission, reoperation, pain, misdiagnosis, disagreement with clinical plan, unclear clinical plan, or delay in care. Reviews that directly referenced a surgical aspect of care were additionally classified as operative. Reviews that could not be classified according to the above criteria were excluded (e.g., “This doctor is a disgrace to his profession!”). In addition, any one-star review that was overwhelmingly positive was also excluded (e.g., “Doctor is an amazing physician. I have seen him over the past 10 years and he has taken excellent care of me”). Reviews were classified and categorized by two separate authors with a third author (ER) to resolve conflicting classifications or categorization between the two reviewers (95.46% agreement and Cohen's kappa statistic of 0.667).

Categorical variables were analyzed using the chi-square test with an alpha set to 0.05. The rate ratio (the ratio of the rate for nonsurgical reviews divided by surgical reviews) was determined for each category. Inter-rater reliability was calculated between the reviewers utilizing Cohen's kappa statistic. A statistical analysis was performed using a commercially available software package (Microsoft Excel, Redmond, WA).

## 3. Results

**Figure 1:**
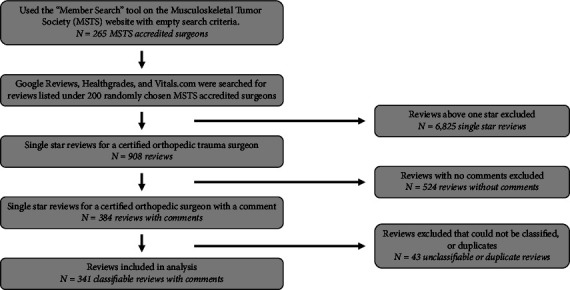
Flowchart of inclusion and exclusion criteria for this study.

A total of 7,733 reviews were discovered. Of these, 908 (11.7%) were identified as one-star reviews. A total of 524 reviews (57.7% of one-star reviews) were excluded as they did not contain any comments to be classified. Of the 384 one-star reviews with comments, 19 were excluded as they were duplicate reviews, 20 were unclassifiable, 2 were positive reviews, and 2 were for the wrong specialty. This left 341 one-star reviews for classification. In total, these 341 one-star reviews comprised a total of 907 complaints. Each review had an average of 2.57 complaints (Figure 1).

### 3.1. Rating

Of the 200 oncology orthopedic surgeons included, 183 (91.5%) were rated at least once. Google.com was the least utilized website for reviews, with 138 (69%) surgeons having at least one review. The average online rating was 4.44 out of 5 for all surgeons (Table 1). The average number of reviews per surgeon was 38.7, and the average number of one-star reviews per physician was 4.54.

### 3.2. Clinical vs. Nonclinical

Of the 907 total complaints, 362 (40.8%) were clinically related, while 545 (59.2%) were nonclinical in nature. Of the 341 one-star reviews, 120 (35.2%) were from surgically treated patients, and 221 (64.8%) were from nonsurgical patients. Surgical patients had a significantly higher rate of clinical complaints than nonsurgical patients (1.89 vs. 0.61 clinical complaints per review, *p* < 0.001). Conversely, nonsurgical patients had a significantly higher rate of nonclinical complaints than surgical patients (1.42 vs. 1.70 nonclinical complaints per review, *p* < 0.001). Nonsurgical patients had a significantly higher rate of total complaints per one-star review than surgical patients (1.76 vs. 0.78 nonclinical complaints per review, *p*=0.034) (Table 2).

### 3.3. Reasons for Negative Review

Clinical factors that were mostly addressed included complications (87 reviews, 26%), disagreement with plan (89 reviews, 26%), uncontrolled pain (59 reviews, 17%), reoperation (37 reviews, 11%), and perceived misdiagnosis (37 reviews, 11%). The most common nonclinical complaints referenced bedside manner (220 reviews, 65%), limited time with the provider (66 reviews, 19%), phone communication issues (57 reviews, 17%), rude or unprofessional conduct (52 reviews, 15%), waiting time (47 reviews, 14%), and scheduling issues (49 reviews, 14%) (Table 3).

### 3.4. Surgical vs. Nonsurgical

Surgical patients comprised a total of 120 reviews. The most common clinical complaints for surgical patients were complication (80 complaints, 67%), uncontrolled pain (43 complaints, 36%), disagreement with the plan (42 reviews, 35%), and reoperation (34 complaints, 28%). The most common nonclinical complaints were about the physician's bedside manner/unprofessionalism (73 complaints, 61%), time spent with the provider (29 complaints, 24%), waiting time (18 complaints, 15%), and phone issues (15 complaints, 13%).

Nonsurgical patients were responsible for 221 reviews. The most common clinical complaints for nonsurgical patients were disagreement with a clinical decision (47 complaints, 21%), misdiagnosis (27 complaints, 12%), delay in care (19 complaints, 9%), and pain (16 complaints, 7%). The most common nonclinical complaints were regarding physicians' bedside manner/unprofessionalism (147 complaints, 67%), phone issues (42 complaints, 19%), scheduling issues (41 complaints, 19%), rude staff (40 complaints, 18%), and time spent with providers (37 complaints, 17%).

The difference in the number of complaints for surgical vs. nonsurgical patients was statistically significant (*p* < 0.05) for multiple complaints (Table 4).

## 4. Discussion

Though many orthopedic surgeons believe that one-star reviews are common on PRWs [10], our study showed that one-star reviews make up only 11.7% of the 7,733 reviews submitted for orthopedic oncologists on Google Reviews, Healthgrades, and Vitals.com. Nonclinical complaints were approximately 1.5 times more common than clinical complaints in one-star reviews of orthopedic oncology surgeons. The most frequent nonclinical complaints were related to physicians' bedside manner, followed by limited time with the provider and phone communication issues. The most common clinical complaints included complications, disagreement with the plan, and uncontrolled pain. These findings are similar to those of other studies in which nonclinical complaints were more common than clinical complaints [15–17].

While physicians rely heavily on clinical outcomes to guide clinical practice and goal-directed improvements, research suggests that patients rely on nonclinical factors in determining the overall quality of their care. A survey investigating patients' perspectives of physicians showed that patients' views of physicians are more heavily influenced by the doctor-patient relationship than health outcomes [18]. As reflected in our study, the single most frequent complaint was in regard to physician bedside manner, which was present in 65% of the 341 reviews studied. These findings have been echoed by numerous other studies examining negative reviews in medicine. Importantly, prior studies have shown that improvement in physician bedside manner is positively correlated with patient satisfaction [19, 20]. Nonclinical complaints regarding phone communication issues have also been previously reported as a common source of patient complaints about physicians [21–23]. Similar to bedside manner, quality of communication has a larger impact on patient satisfaction than quality of care as well [8]. These studies emphasize the results from our study, suggesting that physicians can dramatically improve patient satisfaction through improvements in bedside manner, open communication, and access through telephone encounters.

In our study, complaints of not enough time spent with the provider were second to bedside manner. A study that reviewed 2,185 reviews of orthopedic surgeons from four PRW sites found that time spent with the patient was one of the five factors that was statistically significant in predicting online physician ratings [24]. Another study analyzed 712 online reviews from two rating websites for primary care doctors and found that the majority of Internet reviews of the primary care physicians were positive, but the care encounter extended beyond the physician-patient experience and was dependent on staff, access, and convenience, including shorter wait times [22]. In combination with our study, these results suggest that orthopedic oncologists could better connect with their patients and further reduce one-star reviews and complaints by increasing time spent with patients and thoroughly answering questions during patient encounters. While orthopedic oncologists have limited time due to complicated patients, busy clinic schedules, and various other commitments, physicians who ensure patients feel addressed may benefit from higher online scores and more positive reviews.

Another aspect of the care provided by surgeons that must be considered when attempting to understand negative reviews is the role of the staff who represent the practice. A recent study by Manning et al. reviewed patients' perspectives of midlevel providers in orthopedic sports medicine and found that 62.9% of patients thought a physician's midlevel provider is an important consideration when choosing a physician [25]. Another study analyzed 11,527 reviews by patients of total joint arthroplasty surgeons and revealed significant correlation between physician ratings and staff friendliness, punctuality, and knowledge/expertise [26]. These results suggest that a physician's online reputation is heavily influenced by all members of the physician's team, such as nurses, office staff, medical assistants, and midlevel providers. In this study, we also found that 4% of complaints were related to midlevel providers' bedside manner and 15% were due to rude/unprofessional staff. Multiple reviews included complaints about ancillary staff such as surgery schedulers that negatively impacted the patients' experiences with the orthopedic clinic. For example, one of the reviews read, “I met with (the doctor) who was pleasant, professional, and seemed quite competent. My problem was with the office staff, (the scheduler) in particular. I was strung along for a month waiting for my procedure to be scheduled.” Although surgeons should focus on maintaining strong physician-patient relationships, it is important to acknowledge the contributions of staff in representing the clinic and physician themselves. These examples accentuate why physicians should encourage pleasant staff interactions and appropriate office response time to phone calls to improve patient trust.

Although nonclinical complaints were more common, clinical complaints still comprised 40.8% of all reviews. The three most common clinical complaints were complications, disagreement with a decision/plan, and uncontrolled pain. Complications, such as infection, are not uncommon in orthopedic surgery, particularly in oncology, given the complexity in patients and procedures. As reported, prevalence of infection after orthopedic surgery ranges from 0.7 to 22.7% [27, 28]. However, a study of 1,304 patients who underwent orthopedic oncology procedures for primary bone tumors had an infection rate of 10.1% [29]. Surgical site infection can lead to readmissions, reoperations, and increased costs. Unfortunately, complications are an inherent risk of all surgical procedures. However, not all complications should necessarily result in negative online reviews. One way to improve patient satisfaction despite complications is to clearly communicate risks and benefits while providing expectation management by the physician prior to surgery. In order to improve communication and increase successful medical encounters in orthopedics, the American Academy of Orthopedic Surgeons (AAOSs) created information statements to encourage providers to sit down during patient encounters, show empathy and respect, and involve patients in decisions concerning their medical care [30] as is supported in other resources as well [31, 32]. These adjustments could help orthopedic oncologists improve patient satisfaction through patient autonomy and managing patient expectations.

Our study found that complaints varied greatly depending on whether patients underwent surgical intervention or not. In our study, patients who had a surgical aspect to their care accounted for 35% of one-star reviews, while nonsurgical patients accounted for 65% of one-star reviews. Furthermore, the only nonclinical complaints more common among surgical patients than nonsurgical patients were the lack of time spent with the provider and that wait times were too long. Providers should keep in mind that there is a higher risk of patients leaving one-star reviews if they do not undergo surgical care [33]. The exact reasons for this association are unclear, but it may represent the follow-up with surgical patients that leads to a longer and potentially stronger relationship. Future studies are necessary to fully understand the relationship between surgical care and patient satisfaction.

The accuracy of information provided in online review and rating websites for representing quality of care remains controversial. There have been prior attempts to better understand the dynamics between online reviews and readmission rates, infections, mortality, and morbidity. However, the results among studies have been inconsistent or have shown poorly supported correlations between online ratings and objective health care outcome measures [19, 34, 35].

In addition, credibility is questioned as PRW reviews can be submitted anonymously in a way that cannot be verified [10, 36]. As these PRW sites allow patients to autonomously comment in this public space, any person can create an account and protect their identity.

A survey by C. S. Mott Children's Hospital revealed that two thirds of parents thought some online reviews were “fake” and were not reliable to influence clinical decisions [37]. There are also concerns that some reviews are intentional defamation and unjustified criticism of the physician. In our study, only 1.2% of reviews were classified as defamation without constructive criticism for the surgeon or physician's office. Surgeons should consider that many of the one-star reviews contain constructive criticism helpful to their job performance, while the minority are baseless complaints.

## 5. Limitations

This study has several limitations, the first being that there are many PRWs available. While we have three of the more commonly used PRWs (Google Reviews, Healthgrades, and Vitals.com), our data represent only the PRWs we sampled from. Therefore, negative reviews could present with different compositions and frequencies among different review websites. In addition, it has been noted that only extremely satisfied or extremely dissatisfied patients are likely to write reviews. In effect, patients are self-selected to leave negative reviews on physician review websites. In contrast, it has been reported that fewer negative reviews are submitted for providers who initiate reviews with their patients by providing their own surveys [36]. Given this information, it is possible that patients who write extremely negative reviews might not portray an accurate representation of surgeons' practices. In addition, some patients might be extremely dissatisfied but not submit a review to Google Reviews, Healthgrades, or Vitals.com. Also, it is possible that the reviews evaluated in this study were incorrectly categorized. However, this probability was discarded as two authors independently categorized the variables and a third author resolved any disparities.

Another limitation to discuss is the fact that we chose to only include one-star reviews from the sampled websites instead of including two-star reviews in addition to one-star reviews. While this would increase the number of reviews, we chose to focus only on one-star reviews in order to serve as a control and standardization across websites. Though two-star or three-star reviews may be considered negative, that is not universal across websites. For example, Vitals.com provides a three-star scoring system where a three-star review is considered “good” and thus it could be inferred that a two-star review from Vitals.com is not of equal negativity to a two-star review from a website that uses a wider range scoring system. Narrowing the study to include only one-star reviews was chosen to serve as a control to focus on only the most negative reviews to emphasize the purpose of our study.

## 6. Conclusion

Our study of extremely negative reviews of orthopedic oncology surgeons gathered from Google Reviews, Healthgrades, and Vitals.com found that most complaints were nonclinical in nature. The most common clinical complaints were complications, disagreement with the plan, and uncontrolled pain. Approximately one-third of one-star reviews were by patients who had undergone surgery. In our study, the most common complaints were about physician bedside manner, followed by not enough time spent with the provider, phone and communication issues, waiting time, and scheduling issues. Patients who underwent surgery were more likely to have clinical complaints, the most common being complications, disagreement with treatment plan, and uncontrolled pain. An understanding of factors contributing to negative online reviews can provide the feedback necessary for orthopedic oncology surgeons to improve patient satisfaction and increase trust between physicians and patients. These results reinforce the notion that orthopedic oncology surgeons can improve patient satisfaction with improved communication and an overall positive clinic experience.

## Figures and Tables

**Table 1:** Physician rating website, number of oncology surgeons rated per site, and average score.

Physician review website	No. (%)	Average rating (scale 1–5)
Vitals.com	152 (76%)	4.28
Healthgrades.com	168 (84%)	4.35
Google.com	138 (69%)	4.63
Overall	183 (92%)	4.44

**Table 2:** Total and average number of complaints per review.

	Overall	Surgical (*n* = 120 patients)	Nonsurgical (*n* = 221 patients)	Rate ratio (nonsurgical/surgical complaints)	*P*
Total number of clinical complaints	362	227	135	0.59	<0.001
Average number of clinical complaints per review	0.93	1.89	0.61	—	
Total number of nonclinical	545	170	375	2.21	<0.001
Average number of nonclinical complaints per review	1.52	1.42	1.70	—	
Total complaints	907	397	510	1.28	<0.001
Average number of total complaints per review	2.66	1.16	1.50	—	

**Table 3:** One-star reviews of orthopedic oncology surgeons.

Clinical	No. of reviews (*n* = 341 patients)	% (*n* = 341)
Complication	87	26
Readmission	4	1
Reoperation	37	11
Uncontrolled pain	59	17
Misdiagnosis	37	11
Disagree with decision/plan	89	26
Unclear treatment plan	23	7
Delay in care	26	8

*Nonclinical*		
Bedside manner doctor/unprofessional	220	65
Bedside manner midlevel/unprofessional	13	4
Rude/unprofessional staff	52	15
Wait time	47	14
Not enough time spent with provider	66	19
Cost	14	4
Billing/insurance	20	6
Facilities	4	1
Scheduling issues	49	14
Commute/travel	3	1
Phone communication issues	57	17

**Table 4:** One-star reviews surgical vs. nonsurgical patients of orthopedic oncology surgeons.

Clinical	No. of surgical complaints	Percentage of surgical (%)	No. of nonsurgical complaints	Percentage of nonsurgical complaints (%)	Rate ratio (nonsurgical/surgical)	*P*
Complication	80	67	7	3	0.09	**<0.001**
Readmission	1	1	3	1	3.00	0.824
Reoperation	34	28	3	1	0.09	**<0.001**
Uncontrolled pain	43	36	16	7	0.37	**<0.001**
Misdiagnosis	10	8	27	12	2.70	0.712
Disagree with decision/plan	42	35	47	21	1.12	**<0.001**
Unclear treatment plan	10	8	13	6	1.30	**0.049**
Delay in care	7	6	19	9	2.71	0.746

*Nonclinical*						
Bedside manner doctor/unprofessional	73	61	147	67	2.01	**0.131**
Bedside manner midlevel/unprofessional	3	3	10	5	3.33	0.569
Rude/unprofessional staff	12	10	40	18	3.33	**0.254**
Wait time	18	15	29	13	1.61	**0.088**
Not enough time spent with provider	29	24	37	17	1.28	**0.001**
Cost	4	3	10	5	2.50	0.936
Billing/insurance	6	5	14	6	2.33	0.943
Facilities	0	0	4	2	N/a	0.196
Scheduling issues	8	7	41	19	5.13	**0.028**
Commute/travel	2	2	1	5	0.50	**0.014**
Phone communication issues	15	13	42	19	2.80	0.552

## Data Availability

The data that support the findings of this study are openly available in Google Reviews, Healthgrades, and Vitals.com.
